# Calcified Catheter-Related Fibrin Sheath Forms Large Intravenous Cast in Hemodialysis Patient Causing Embolic Sequelae

**DOI:** 10.7759/cureus.31714

**Published:** 2022-11-20

**Authors:** Justin Newman, Ali Syed, Chava Blivaiss, Joshua Melamed, Shahriyour Andaz

**Affiliations:** 1 Medical Student, New York Institute of Technology (NYIT) College of Osteopathic Medicine, Glen Head, USA; 2 Medical Student, Medical College of Wisconsin, Milwaukee, USA; 3 Thoracic Surgery, Mount Sinai South Nassau, Oceanside, USA; 4 Cardiothoracic Surgery, Medical College of Wisconsin, Milwaukee, USA

**Keywords:** right atrium, calcification, catheter-tip position, general nephrology dialysis and transplantation, methicillin resistant staphylococcus aureus (mrsa), catheter related sheath

## Abstract

Catheter-related sheath (CRS) formation secondary to chronic indwelling central venous catheters (CVC) is a well-documented complication. When these fibrin sheaths calcify, they can form a “cast” surrounding the catheter. Upon removal of the CVC, a rare complication can occur where the calcified sheath remains in situ leaving behind an intraluminal catheter-shaped cast. This report describes a case of a 57-year-old dialysis-dependent woman who was found to have a right internal jugular vein cast during the evaluation and treatment of methicillin-resistant Staphylococcus aureus (MRSA) bacteremia. This case reviews and discusses the embolic complications suspected to be a result of this cast. Our case provides insight into the clinical course, diagnostic methods, and imaging identification of a rare pathology and its unique complications.

## Introduction

More than five million central venous catheters (CVCs) are inserted in the United States annually, with approximately 8% of hospitalized patients requiring central venous access [1,2]. Prolonged use of CVCs is associated with delayed complications including but not limited to infection, thrombosis, pulmonary emboli, venous stenosis, and catheter malfunctions. Fibrin sheath thrombi, or catheter-related sheaths (CRS), are a common complication of chronic indwelling CVCs. Fibrin sheaths result from thrombus formation and endothelial cell damage due to catheterization that stimulates the activation of smooth muscle cells (SMC). SMC migration into the peri-catheter thrombus transforms it into collagen tissue termed a sheath that can calcify [3-5]. Enough calcification can generate a “cast” around the catheter which can be left behind when the catheter is removed. This is usually discovered incidentally on an x-ray, transthoracic echocardiogram, or computed tomography (CT) scan. This report presents a patient with an incidental diagnosis of a right internal jugular vein calcified cast extending into the superior vena cava (SVC) and right atrium (RA). Additional findings are suggestive of catheter or cast fragments and septic emboli.

This article was previously posted to the ResearchSquare preprint server on May 18th, 2021 [6]. 

## Case presentation

A 57-year-old female with end-stage renal disease on dialysis, type II diabetes mellitus, hypertension, hyperlipidemia, and spina bifida presented to the emergency department for the evaluation of possible catheter-related infection after a positive outpatient methicillin-resistant Staphylococcus aureus (MRSA) blood culture. The patient had been receiving dialysis through CVCs for several years after two failed attempts at arteriovenous fistulas (AVF) and refusal of reattempts. Within the past ten months, the patient had been seen in the hospital for bacteremia twice. Three months prior to the presentation, the patient was treated in the hospital for coagulase-negative bacteremia and discharged from the hospital with a plan to complete eight weeks of intravenous vancomycin.

**Figure 1:**
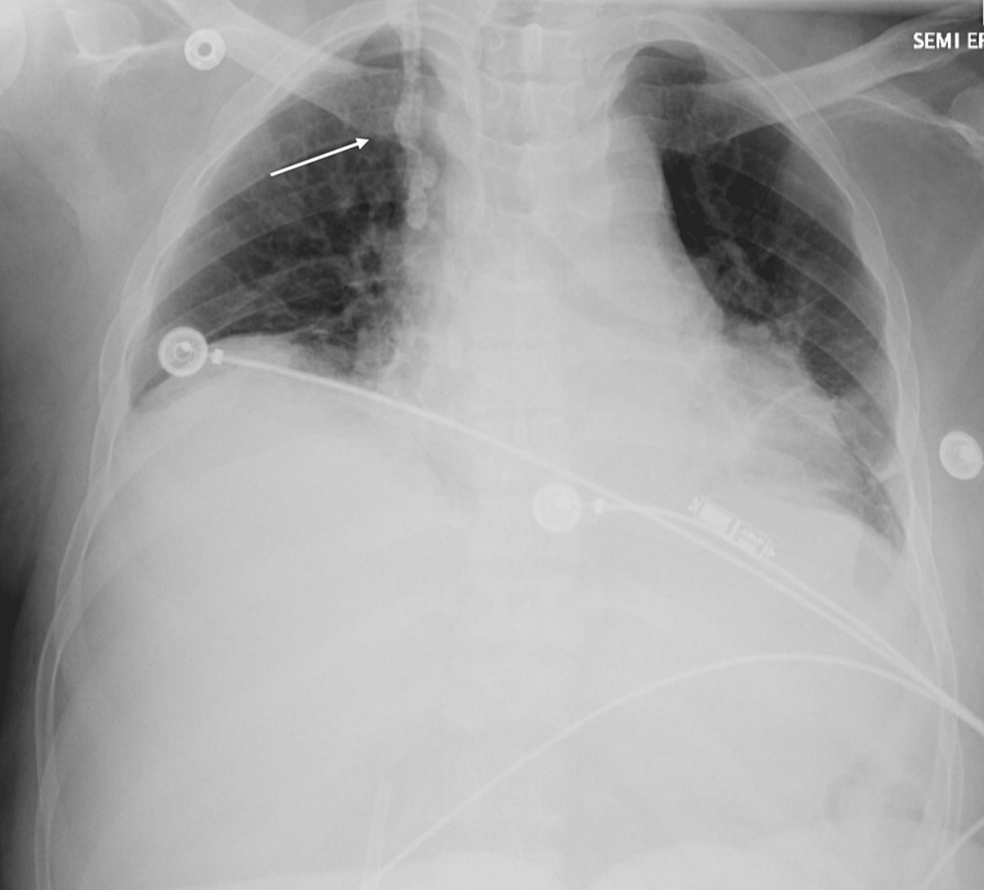
Chest x-ray demonstrating calcific density projecting over the right lower neck

On admission, the patient voiced no complaints and denied fevers, chills, sick contacts, headache, shortness of breath, chest pain, and abdominal pain. The patient’s vital signs were within normal limits. A physical exam revealed a tender right groin along the path of a right femoral tunneled dialysis catheter (TDC) though no erythema, fluctuance, or expressible purulence was present. Bloodwork indicated anemia, normal white blood cell count, and mild thrombocytopenia. Metabolic studies revealed uremia and hyperphosphatemia. After consultation with an infectious disease specialist, the patient was started on daptomycin. Chest radiographs obtained in the emergency department detailed a stable, vertically oriented calcific density projecting over the right lower neck and right lung apex (Figure 1).

The vascular surgery department consulted with the patient and decided to exchange the right femoral TDC because it was believed to be the nidus for infection. Guidewire exchange was unsuccessful resulting in the removal of the right femoral TDC with a plan for fluoroscopy-guided catheter placement the next day. The catheter was sent for pathology but would eventually yield no growth. A transthoracic echocardiogram was ordered for the evaluation of endocarditis, though no vegetation was visualized. A right femoral vein TDC was situated under fluoroscopy guidance.

**Figure 2:**
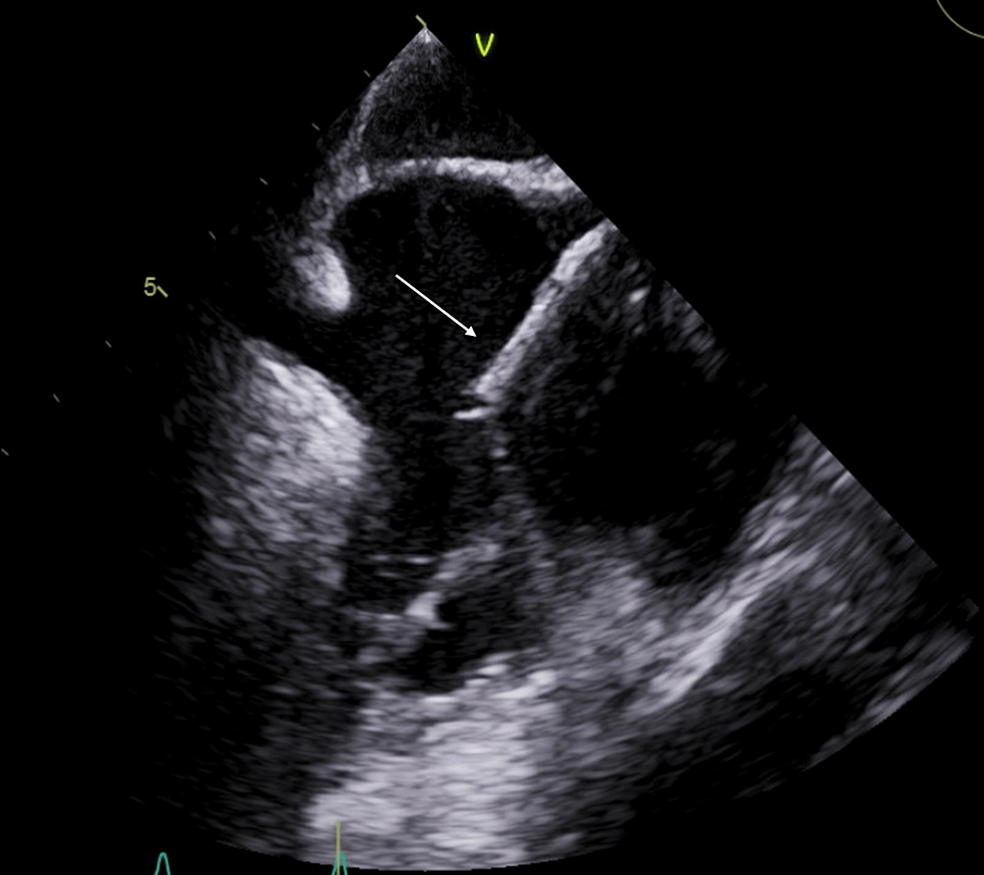
Transesophageal echocardiogram bicaval view. The image shows a calcified cast in the super vena cava entering the right atrium.

**Figure 3:**
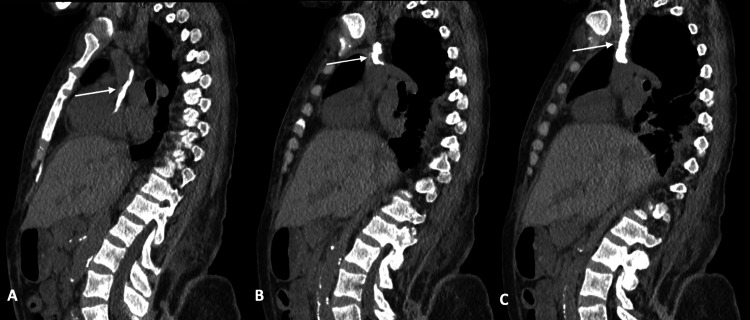
Noncontrast sagittal CT images from left lateral to right (A-C). Images demonstrate a right internal jugular vein calcified cast with its distal tip in the right atrium.

**Figure 4:**
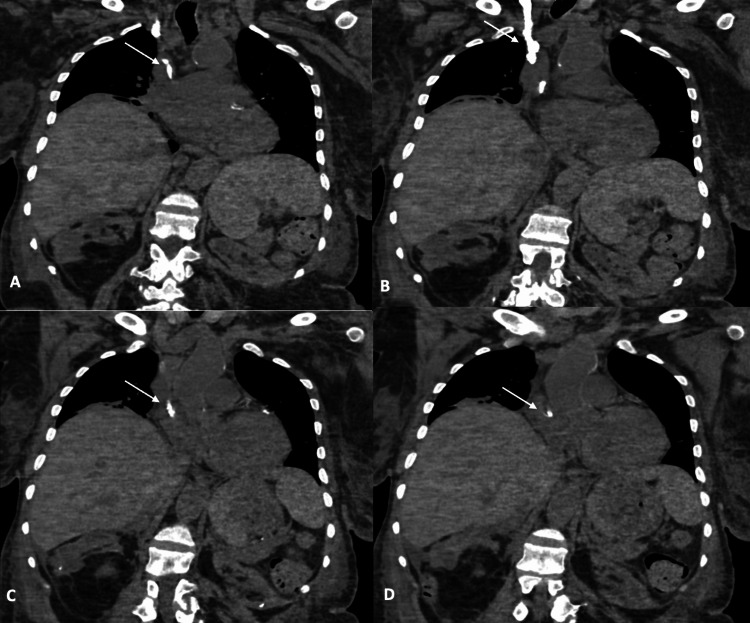
Non-contrast coronal CT images from anterior to posterior (A-D). Images show an irregular, linear, cylindrical radio-opaque density in the right internal jugular vein.

Follow up transesophageal echocardiogram (TEE) was ordered and showed a catheter within the SVC entering the RA (Figure 2). There was no explanation for this finding in the patient’s history. Thoracic surgery was consulted to evaluate what appeared to be a catheter tip remnant in the SVC and RA. A CT scan of the chest without contrast showed a chronically calcified right internal jugular “central venous catheter” with the distal tip located in the RA (Figures 3, 4). Band-like areas of subcutaneous atelectasis were noted with additional patchy areas of peripheral consolidation reflective of septic emboli. Operative intervention for the atrial foreign body was considered but ruled out in favor of medical management and the patient was discharged on doxycycline indefinitely.

 

## Discussion

Calcifications of CRSs are uncommon. In one retrospective study, 147 adults underwent CT after CVC removal, and the prevalence of fibrin sheath remnants was found to be 13.6%, of which 45% were calcified [5]. In a literature review published by Matusik et al., only eight case reports of retained calcified fibrin sheaths diagnosed after dialysis CVC removal have been published. Embolic sequelae of these casts are rare. Only one case describing the embolization of a cast has been documented** **[7]. This patient represents an opportunity to expand our clinical knowledge and understanding of this rare clinical complication.

**Figure 5:**
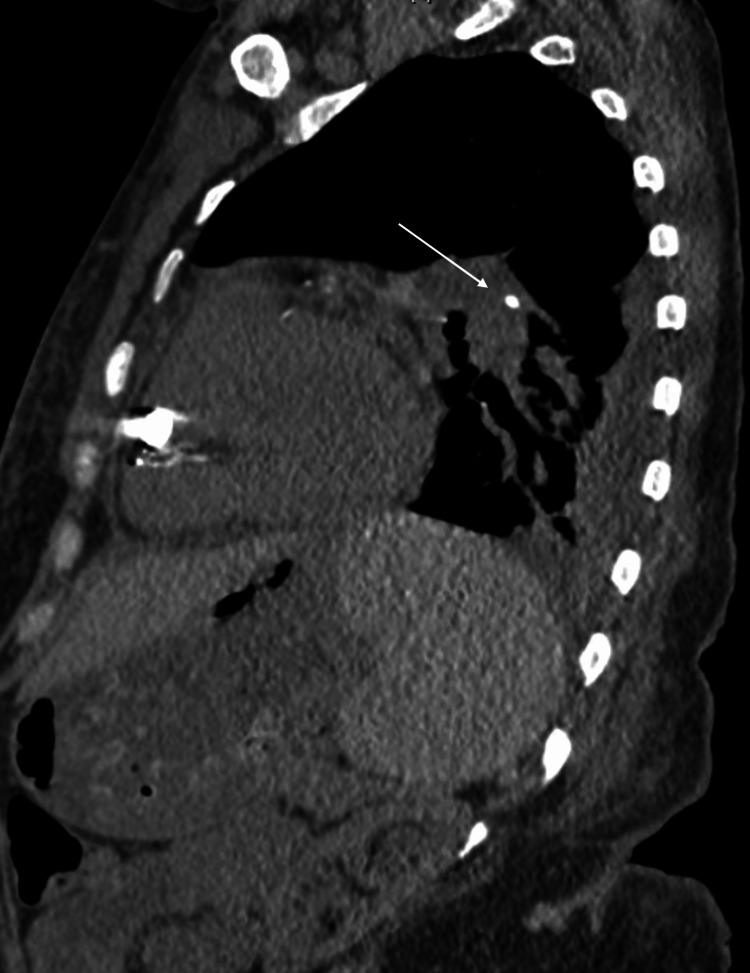
Non-contrast sagittal chest CT demonstrates calcific embolism within the left pulmonary artery.

**Figure 6:**
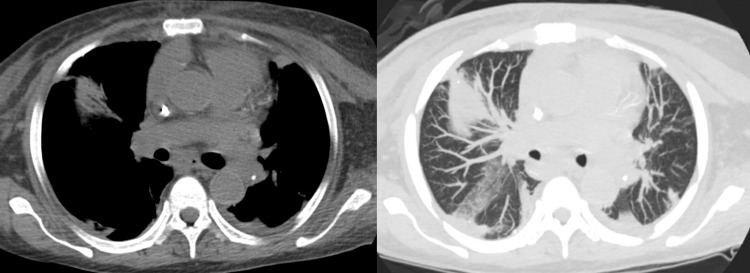
(Left) Noncontrast axial CT demonstrating left pulmonary artery embolism with cast visualized in the superior vena cava. (Right) Maximum intensity projection view demonstrates same findings.

This patient’s CT findings deviate from the more commonly described “tubular” casts discussed in the literature [5]. CT findings in our patient can be described as an irregular, linear, cylindrical radio-opaque density. CT imaging of our patient is suggestive of embolization of either a broken calcified catheter tip or the tip of the cast alone. The embolic substance can be seen on CT resting within the left pulmonary artery (Figures 5, 6). Furthermore, we believe that either cast fragments and/or septic emboli were being shed into the distal pulmonary vasculature. In this patient, the cast was likely acting as a nidus for infection provided that the femoral TDC pathology was unremarkable. Rousslang et al. described a similar case in which a patient ten months post-CVC removal suffered cast embolization into the right pulmonary artery [8].

Patients with end-stage renal disease on hemodialysis are more prone to casts due to abnormal calcium and phosphate metabolism [9,10]. The most common risk factors contributing to cast formation in dialysis patients are pro-thrombotic and pro-calcification factors related to patient comorbidities and prolonged catheter dwell time [7]. While our patient remained borderline hypocalcemic throughout her hospital stay, phosphate was markedly elevated. Blood urea was notably elevated contributing to a pro-thrombotic state, though the dwell time of the CVC in our patient was unknown [9]. The precise CVC that caused this patient’s complications can’t be discerned, and therefore it is likely that this calcification process went unnoticed.

Our patient remained asymptomatic and hemodynamically stable over the course of her stay and thus medical management with antibiotics and anticoagulation was favored over surgical intervention. Management of ectopic calcification can include the moderation of dietary calcium and phosphorus as well as non-calcium phosphorus binding agents [9]. Serial imaging should be utilized to monitor cast progression in patients. This case makes evident that chest x-rays can demonstrate casts, though clinical suspicion would need to be high for further workup. CT and TEE remain superior modalities for visualization. Calcified catheter casts should be included in the differential diagnosis when irregular radiopaque findings are seen in the distribution of a CVC before or after explanation with a history of central line placement.

## Conclusions

Our patient remained asymptomatic and hemodynamically stable over the course of her stay and thus medical management with antibiotics and anticoagulation was favored over surgical intervention. Management of ectopic calcification can include the moderation of dietary calcium and phosphorus as well as non-calcium phosphorus binding agents. Serial imaging should be utilized to monitor cast progression in patients. This case makes evident that chest x-rays can demonstrate casts, though clinical suspicion would need to be high for further workup. CT and TEE remain superior modalities for visualization. Calcified catheter casts should be included in the differential diagnosis when irregular radiopaque findings are seen in the distribution of a CVC before or after explanation with a history of central line placement.
